# De novo production of the flavonoid naringenin in engineered *Saccharomyces cerevisiae*

**DOI:** 10.1186/1475-2859-11-155

**Published:** 2012-12-08

**Authors:** Frank Koopman, Jules Beekwilder, Barbara Crimi, Adele van Houwelingen, Robert D Hall, Dirk Bosch, Antonius JA van Maris, Jack T Pronk, Jean-Marc Daran

**Affiliations:** 1 Department of Biotechnology, Delft University of Technology, Julianalaan 67, 2628 BC, Delft, the Netherlands; 2 Platform for Green Synthetic Biology, P.O. Box 5057, 2600 GA, Delft, The Netherlands; 3 Kluyver Centre for Genomics of Industrial Fermentation, P.O. Box 5057, 2600 GA, Delft, The Netherlands; 4 Plant Research International (PRI), P.O. Box 16, 6700 AA, Wageningen, The Netherlands; 5 Centre for Biosystems Genomics, PO Box 98, 6700 AB, Wageningen, The Netherlands

**Keywords:** *Saccharomyces cerevisiae*, Naringenin, *de novo*, Flavonoids, Metabolic engineering

## Abstract

**Background:**

Flavonoids comprise a large family of secondary plant metabolic intermediates that exhibit a wide variety of antioxidant and human health-related properties. Plant production of flavonoids is limited by the low productivity and the complexity of the recovered flavonoids. Thus to overcome these limitations, metabolic engineering of specific pathway in microbial systems have been envisaged to produce high quantity of a single molecules.

**Result:**

*Saccharomyces cerevisiae* was engineered to produce the key intermediate flavonoid, naringenin, solely from glucose. For this, specific naringenin biosynthesis genes from *Arabidopsis thaliana* were selected by comparative expression profiling and introduced in *S. cerevisiae.* The sole expression of these *A. thaliana* genes yielded low extracellular naringenin concentrations (<5.5 μM). To optimize naringenin titers, a yeast chassis strain was developed. Synthesis of aromatic amino acids was deregulated by alleviating feedback inhibition of 3-deoxy-d-arabinose-heptulosonate-7-phosphate synthase (Aro3, Aro4) and byproduct formation was reduced by eliminating phenylpyruvate decarboxylase (Aro10, Pdc5, Pdc6). Together with an increased copy number of the chalcone synthase gene and expression of a heterologous tyrosine ammonia lyase, these modifications resulted in a 40-fold increase of extracellular naringenin titers (to approximately 200 μM) in glucose-grown shake-flask cultures. In aerated, pH controlled batch reactors, extracellular naringenin concentrations of over 400 μM were reached.

**Conclusion:**

The results reported in this study demonstrate that *S. cerevisiae* is capable of *de novo* production of naringenin by coexpressing the naringenin production genes from *A. thaliana* and optimization of the flux towards the naringenin pathway. The engineered yeast naringenin production host provides a metabolic chassis for production of a wide range of flavonoids and exploration of their biological functions.

## Background

In recent years, plant flavonoids, which comprise a family of over 9000 compounds, have attracted a tremendous increase in research interest [1-3]. This interest is mainly attributed to highly promising human health applications of specific flavonoids [4-8]. The biological activities of flavonoid compounds have been investigated in relation to a multitude of human pathological conditions, including cancer, diabetes, obesity and Parkinson’s disease [6,9-14]. The identified mechanisms of action include scavenging of oxygen radicals, anti-inflammatory, antiviral and antitumor activities [15,16].

Both for health related research and commercial nutritional applications, availability of sufficient amounts of defined flavonoid preparations is important. To date, flavonoid production mostly relies on isolation from plants. However, investigation and subsequential industrialization from plants is hampered by their low production efficiency. In addition to the low growth rate of some of the producing plants, extraction and separation of flavonoids with highly related structures complicate plant-based production, thereby impeding progress in the exploration of the biological activities of flavonoids [13,14]. Although flavonoids can be produced chemically, efficient production of flavonoids by organic synthesis is severely hindered by the complexity of the molecules, as well as by the necessity of utilizing toxic chemicals and extreme reaction conditions [13,14].

**Figure 1:**
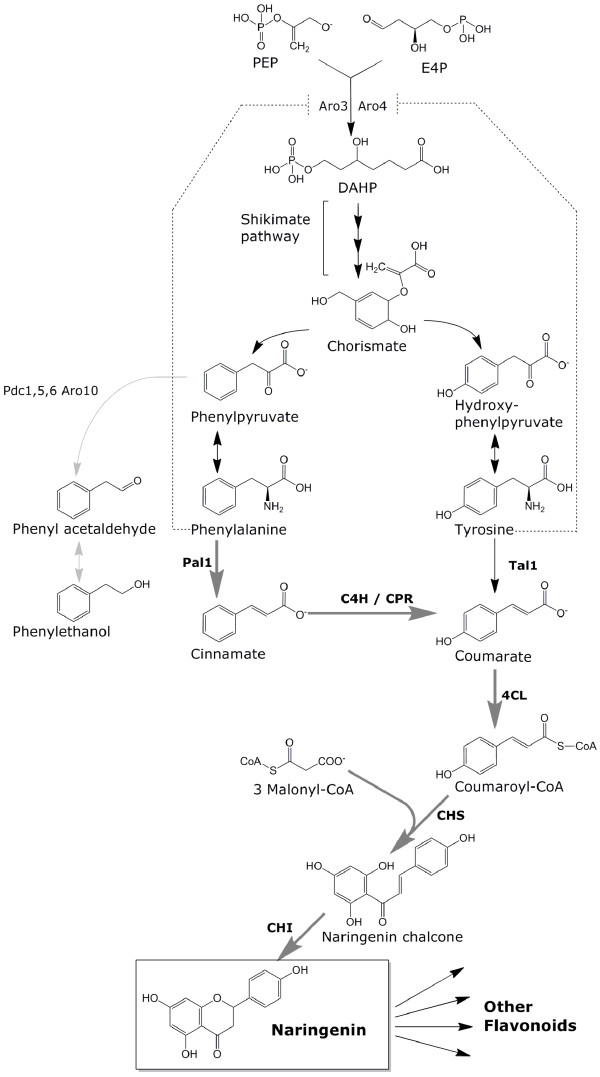
**Schematic representation of the engineered naringenin production pathway in*S. cerevisiae.*** Six *A. thaliana* genes were overexpressed: *PAL1* (phenylalanine ammonia lyase), *C4H* (Cinnamate 4-hydroxylase), *CPR1* (cytochrome P450 reductase), *4CL3* (4-coumaric acid-CoA ligase), *CHS3* (chalcone synthase) and *CHI1* (chalcone isomerase), and one gene from *Rhodobacter capsulatus*; Tal1 (tyrosine ammonia lyase). Dashed lines indicate feedback inhibition. Grey arrows indicate the *S. cerevisiae* pathway for phenylethanol production. Bold dark grey arrows indicate the naringenin production pathway as described for *A. thaliana*[22]*.* Aro3/Aro4: 3-deoxy-D-arabino-heptulosonate-7-phosphate (DAHP) synthase, Pdc1, 5, 6; pyruvate decarboxylases, Aro10; phenylpyruvate decarboxylase.

In response to the poor production efficiency from plants and chemical synthesis, research groups have directed their attention to the heterologous production of flavonoids in microorganisms such as *Escherichia coli* and *Saccharomyces cerevisiae,* using metabolic engineering and synthetic biology [14,17-20]. In plants, formation of the central metabolite naringenin involves six steps catalyzed by phenylalanine ammonia lyase (*PAL*), cinnamate 4-hydroxylase (*C4H*) and its associated cytochrome P450 reductase (*CPR*), 4-coumaric acid-CoA ligase (*4CL*), chalcone synthase (*CHS*) and chalcone isomerase (*CHI*) (Figure 1) [21,22]. Alternatively, a tyrosine ammonia lyase (*TAL*) can convert tyrosine directly to coumaric acid and circumvent the use of membrane bound P450 related enzymes, which may pose challenges in *E. coli*[14,18]. Additionally, in plants, these enzymes and their corresponding genes are often represented by several isoforms, which may differ in substrate preference or kinetic properties [23]. Moreover, the different isoforms are proposed to be organized in one or more enzyme complexes that may promote substrate channeling. In *Arabidopsis thaliana*, interaction between CHS, CHI and an enzyme downstream of naringenin, flavonol 3-hydroxylase were demonstrated based on affinity chromatography and immunoprecipitation assays [24-26].

Expression of enzyme combinations, originating from a variety of host organisms has yielded microbial strains capable of producing the key flavonoid precursor naringenin (Figure 1). Several reports describe successful biotransformation processes in which a phenylpropanoid precursor, such as coumaric acid, is converted into naringenin by metabolically engineered *E. coli* or *S. cerevisiae*[1,14,17,19,20,27,28]. The highest naringenin titers obtained through biotransformation were achieved in *E. coli*, with naringenin titers reaching 1.74 mM (474 mg·l^-1^) from 2.6 mM coumaric acid supplied to the medium [1]. The reliance on ‘expensive’ phenylpropanoid precursors might represent a major hurdle for economically feasible flavonoid production [18]. Hitherto, only one study reports *de novo* naringenin production from glucose. Using an engineered *E. coli* strain, naringenin titers of up to 106.5 μM (29 mg·l^-1^) were obtained [18].

*S. cerevisiae* has several attractive characteristics as a metabolic engineering platform for flavonoid production. In addition to its excellent accessibility to molecular and synthetic biology techniques [29,30], its eukaryotic nature may facilitate functional expression of plant-derived flavonoid-biosynthetic genes. For example, *S. cerevisiae* can functionally express cytochrome P450-containing enzymes and its subcellular compartmentation is comparable to that of plant cells [31]. Finally, its GRAS (generally recognized as safe) status facilitates subsequent application for the production of pharma- and nutraceuticals.

The goal of the present study was to define a metabolic engineering strategy for *de novo* production of naringenin by S*. cerevisiae*, using glucose as sole carbon source. For optimal synergistic activity, the flavonoid biosynthetic genes *PAL1, C4H, CPR1, 4CL3, CHS3* and *CHI1* used in this study were derived from a single plant species, *A. thaliana* and selected for *in planta* co-expression profiles. After expression of the plant pathway genes, optimization of naringenin production was explored by engineering of precursor supply to the naringenin pathway and by reducing the formation of byproducts derived from yeast metabolism.

## Results

### Selection of naringenin biosynthetic genes from *A. thaliana*

As a first step towards heterologous expression of naringenin in *S. cerevisiae,* flavonoid biosynthetic genes were selected from *A. thaliana*. It has been proposed that, in plants, flavonoid biosynthetic enzymes can be organized in protein complexes, where the proteins involved in the pathway are spatially co-localized. The combination of isoenzymes in such complexes is hypothesized to facilitate the synthesis of different flavonoids [24]. In addition, complex formation might enhance pathway activity and carbon flux via metabolic channeling of intermediates [24]. Moreover, formation of toxic intermediates is kept locally and are sequentially converted [32]. Protein complex formation is likely to require specific protein-protein interactions and therefore, co-evolution of the protein structures of the enzymes that form these complexes. In *A. thaliana*, several isoenzymes exist for most of the reactions leading to naringenin formation (Table 1). In order to identify the best set of isoenzymes for naringenin production and maintain the potential benefit of plant-specific protein-protein interactions upon expression in *S. cerevisiae*, a subset of isoenzymes from one single species, *A. thaliana,* was identified and subsequently subjected to expression profile correlation analysis. The subset of isoenzymes was selected based on previous reports indicating either the involvement in the lignin biosynthetic pathway [33] and/or the flavonoid biosynthetic pathway (Figure 1), since these pathways share the same reactions up to the metabolic branch-point coumaroyl-CoA. The specific genes from this isoenzyme subset were then further assessed for the correlation of their expression levels in a dataset comprising 392 transcriptome studies of *A. thaliana*[34].

**Table 1:** ***A. thaliana *genes included in the expression correlation studies**

**Gene**	**Gene product**	***A. thaliana *locus**
***PAL1***	phenylalanine ammonia lyase	at2g37040
*PAL2*	phenylalanine ammonia lyase	at3g53260
*PAL3*	phenylalanine ammonia lyase	at5g04230
*PAL4*	phenylalanine ammonia lyase	at3g10340
***C4H***	trans-cinnamate 4-monooxygenase	at2g30490
*4CL1*	4-coumaric acid-CoA ligase	at1g51680
*4CL2*	4-coumaric acid-CoA ligase	at3g21240
***4CL3***	4-coumaric acid-CoA ligase	at1g65060
*4CL4*	4-coumaric acid-CoA ligase	at3g21230
*CHS1*	Chalcone synthase	at4g00040
*CHS2*	Chalcone synthase	at4g34850
***CHS3***	Chalcone synthase	at5g13930
***CHI1***	Chalcone isomerase	at5g05270
*CHI2*	Chalcone isomerase	at3g63170
*CHI3*	Chalcone isomerase	at3g55120
*F3H*	Flavanone 3-hydroxylase	at3g51240
*FLS*	Flavonol synthase	at5g08640
*HCT*	Quinate O-hydroxycinnamoyltransferase	at5g48930
*CCoOMT*	Caffeoyl-CoA 3-O-methyltransferase	at4g34050
*C3H*	p-coumaric acid 3-hydroxylase	at2g40890

Bold letters indicate genes used in the construction of engineered *S. cerevisiae* strains.

**Figure 2:**
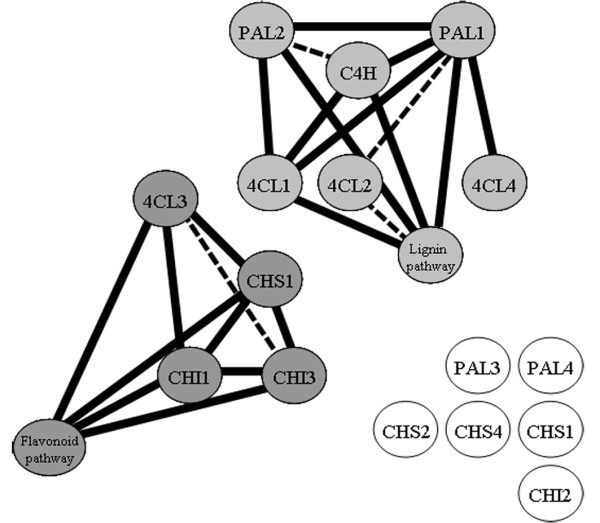
**Co-expression analysis of candidate genes for flavonoid biosynthesis in *A. thaliana*.** Correlations between the expression of a set of target genes (see Table 1) were established by bioinformatic analysis of 392 *A. thaliana* transcriptome datasets [34]. Circles represent genes as described in Table 1. Light grey circles represent genes where the expression correlates with that of genes involved in lignin metabolism (represented by *C3H*, *HCT*, *CCoOMT*), dark grey circles represent genes with expression correlating with genes of the flavonoid pathway (represented by *FLS*, *F3H* and several *UGTs*). White circles represent genes with expression profiles which do not correlate with genes involved in either the lignin or the flavonoid pathway. Solid lines represent Pearson correlations of expression above 0.7, dotted lines represent Pearson correlations between 0.6 and 0.7.

From the expression correlation analysis, using the BAR Expression Angler [35], two clear modules could be distinguished (Figure 2). The first module comprises flavonoid biosynthetic genes, including the 4-coumaric acid-CoA ligase gene (*4CL3*), the chalcone synthase gene (*CHS3*), two chalcone isomerase genes (*CHI1*, *CHI3*) and a number of genes that mediate the further modification of naringenin. The second, a lignin module, comprised two phenylalanine ammonia lyase *(PAL1*, *PAL2*) genes, the trans-cinnamate 4-monooxygenase *(C4H)* gene and three 4-coumaric acid-CoA ligase genes (*4CL1*, *4CL2*, *4CL4*), in addition to a number of genes known to be involved in formation of lignin and phenolic esters.

Since these results are in good agreement with a previous study on expression correlation in plants [36], the following genes for the pathway towards naringenin were selected for heterologous expression in *S. cerevisiae*: the flavonoid biosynthetic genes *4CL3* (at1g65060)*, CHS1* (at4g00040) and *CHI1* (at5g05270), as well as, *C4H* (at2g30490) and *PAL1* (at2g37040) from the lignin biosynthetic genes [37]. *PAL1* and *CHI1* were preferred over *PAL2* and *CHI3*, as their expression profiles showed stronger correlations with *C4H* and *4CL3* respectively (Figure 2).

### Construction and evaluation of a naringenin producing strain

**Figure 3:**
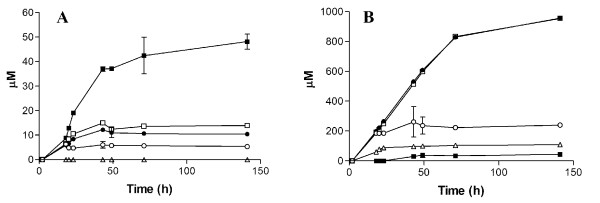
**Naringenin production in*S. cerevisiae.*A**) Increase in heterologous production of naringenin. **B**) Phenylethanol production in engineered strains. (○) IMU011 (*atPAL1*↑, *coC4H*↑, *coCPR1*↑, *atCHI1*↑, *atCHS3*↑_,_*at4CL3*↑), (●) IMX183 (*aro3*Δ *ARO4*^*G226S*^*atPAL1*↑, *coC4H*↑, *coCPR1*↑, *atCHI1*↑, *atCHS3*↑_,_*at4CL3*↑), (□) IMX185 (*aro3*Δ *ARO4*^*G226S*^*pdc5*Δ, *pdc6*Δ, *atPAL1*↑, *coC4H*↑, *coCPR1*↑, *atCHI1*↑, *atCHS3*↑_,_*at4CL3*↑), (■) IMX106 (*aro3*Δ *ARO4*^*G226S*^*aro10*Δ, *pdc5*Δ, *pdc6*Δ, *atPAL1*↑, *coC4H*↑, *coCPR1*↑, *atCHI1*↑, *atCHS3*↑_,_*at4CL3*↑), (Δ) non-naringenin producing reference strain CEN.PK2-1C. Cultures were grown in shake flasks on synthetic medium containing 20 g·l^-1^ glucose and appropriate growth factors to supplement the auxotrophic requirements of the strains. All cultures were performed in triplicate. Error bars denote standard deviation.

To enable naringenin production in *S. cerevisiae*, one episomal and one integrative expression vector were constructed which together, harbor the five flavonoid biosynthetic genes. Additionally, activation of the cytochrome P450 *C4H* requires a cytochrome P450 reductase (*CPR*). To choose the best candidate gene, the two *A. thaliana CPR* variants (*CPR1* or *CPR2*) were separately included in the pathway engineering strategy. First, the centromeric episomal plasmid pUDE172, carrying *PAL1* and yeast codon-optimized versions of the *A. thaliana C4H* (*coC4H*) and *CPR1* (*coCPR1*) genes was constructed. In a same manner, separate construct, *PAL1* and *coC4H* were combined with the *coCPR2* gene. Subsequently, the integration plasmid pUDI065 was constructed, which carried the non yeast optimized *A. thaliana* genes *at4CL3, atCHS1* and *atCHI1*. Only the*, C4H* and *CPR1* in the naringenin production pathway were codon optimized since these genes hold the majority of rare codons for yeast (8), compared to the other genes (*PAL1, 4CL3, CHS3* and *CHI1*) (3). When *coCPR2* was expressed instead of *coCPR1*, significantly lower naringenin titers were observed (data not shown); *coCPR2* was therefore not used in further experiments. Introduction of the *coCPR1* version of the centromeric expression vector and the integration vector yielded the *S. cerevisiae* strain IMU011. In shake-flask cultures containing synthetic medium and with glucose as the sole carbon source, this strain produced naringenin to a concentration of 5.4 μM (Figure 3A). Interestingly, naringenin was measured extracellularly, although its export mechanism has not yet been elucidated.

### Alleviation of tyrosine feedback inhibition of yeast 3-deoxy-D-arabinose-heptulosonate-7-phosphate (DAHP) synthase

Since phenylalanine is a key intermediate in naringenin biosynthesis (Figure 1), further metabolic engineering was directed at improving its intracellular availability. Aromatic amino acid biosynthesis in *S. cerevisiae* is subject to strong feedback inhibition by phenylalanine and tyrosine. Introduction of a tyrosine insensitive *ARO4* allele (*ARO4*^*G226S*^) [38,39] in conjunction with the deletion of the other allele of the 3-deoxy-d-arabinose-heptulosonate-7-phosphate (DAHP) synthase (*ARO3*) has previously been shown to cause a 4-fold increase in flux through the aromatic amino acid pathway [40]. To test the potential impact of this genetic intervention on naringenin synthesis, *S. cerevisiae* strain IMX183 (*ARO4*^*G226S*^*aro3Δ, atPAL1*↑, *coC4H*↑, *coCPR1*↑, *atCHI1*↑, *atCHS3*↑_,_*at4CL3*↑) was constructed. The deregulation of DAHP synthase in this strain lead to a 2-fold increase of the naringenin titer in shake flask cultures (10.4 μM) (Figure 3). However, this increase was accompanied by a strongly enhanced accumulation of phenylethanol, whose extracellular concentration was increased 100-fold to a level ca. 20 fold higher than the improved naringenin concentration (Figure 3B). Therefore, to improve further naringenin production, we adopted a strategy to reduce the diversion of aromatic amino acid biosynthesis into this byproduct.

### Elimination of competing phenylpyruvate decarboxylase activity

**Figure 4:**
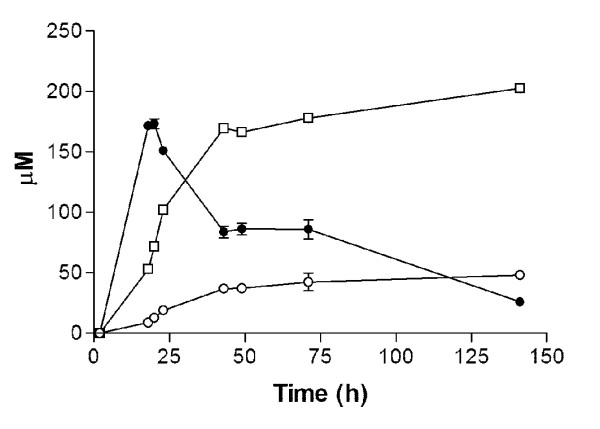
**Product formation by *S. cerevisiae *IMX106 (*aro3*Δ *ARO4***^***G226S***^***aro10*Δ, *pdc5*Δ, *pdc6*Δ, *atPAL1***↑**, *coC4H***↑**, *coCPR1***↑**, *atCHI1***↑**, *atCHS3***↑_**,**_***at4CL3***↑**).** Formation of naringenin (○),coumaric acid (●) and phloretic acid (□). Cultures were grown in shake flasks on synthetic medium containing 20 g·l^-1^ glucose. All cultures were performed in triplicate. Error bars denote standard deviation.

In *S. cerevisiae*, phenylethanol is produced via the Ehrlich pathway. Decarboxylation of phenylpyruvate, the 2-oxo acid associated to phenylalanine, yields phenylacetaldehyde, which is subsequently reduced into phenylethanol and/or oxidized to phenylacetate [41-43]. Decarboxylation of phenylpyruvate can be catalyzed by four different thiamine pyrophosphate-dependent 2-oxo acid decarboxylases encoded by *ARO10, PDC1, PDC5,* and *PDC6*[43,44]. Recent work in our group has demonstrated that, among the four decarboxylases capable of phenylpyruvate decarboxylase, Pdc1 and Pdc6 showed a much lower affinity and decarboxylation rate of phenylpyruvate than Pdc5 and, in particular, Aro10 [44]. Since absence of all three pyruvate decarboxylase genes (*PDC1*, *PDC*5 and *PDC6*) abolishes growth on glucose in synthetic media, strains only retaining *PDC1* (*aro10Δ, pcd5Δ, pdc6Δ*) were constructed [45]. The intermediate strain IMX185 (*ARO4*^*G226S*^, *aro3Δ, pdc5Δ, pdc6Δ*) did not show a reduced phenylethanol titer compared to its ancestor IMX183 (*ARO4*^*G226S*^, *aro3Δ*) (Figure 3B). In contrast, the naringenin producing strain IMX106 (*ARO4*^*G226S*^*, aro3Δ, pdc5Δ, pdc6Δ, aro10Δ*) that also carries the *ARO10* deletion exhibited a 22-fold lower concentration of extracellular phenylethanol (44 μM) compared to both ancestor strains IMX183 and IMX185 (955 μM). This strong reduction in phenylethanol production coincided with a 3-fold increase of extracellular naringenin (up to 46.5 μM) (Figure 3A), indicating that reduction of the flux through the Ehrlich pathway had indeed led to substantial improvement in naringenin production. Analysis of culture supernatants revealed that strain IMX106 also produced coumaric acid up to a concentration of 200 μM (Figure 4). Moreover, this strain showed increased production of another aromatic metabolite that, based on HPLC spectra and LC-MS analysis, was identified as phloretic acid (Figure 4). Production of phloretic acid was only observed in cultures that also produced coumaric acid and which expressed *4CL3* (data not shown). This suggests that phloretic acid is most likely produced from coumaroyl-CoA (Figure 4).

### Alleviate the bottleneck downstream of coumaric acid

**Figure 5:**
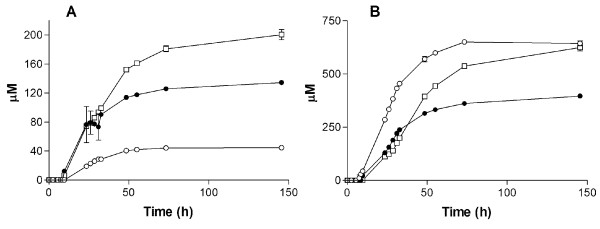
**Stepwise increase in naringenin formation by*S cerevisiae.*** Formation of **A**) naringenin and **B**) phloretic acid in the engineered strains IMX106 (○)(*aro3*Δ *ARO4*^*G226S*^*aro10*Δ, *pdc5*Δ, *pdc6*Δ, *atPAL1*↑, *coC4H*↑, *coCPR1*↑, *atCHI1*↑, *atCHS3*↑_,_*at4CL3*↑), IMX197 (●)(*aro3*Δ *ARO4*^*G226S*^*aro10*Δ, *pdc5*Δ, *pdc6*Δ, *atPAL1*↑, *coC4H*↑, *coCPR1*↑, *atCHI1*↑, *atCHS3*↑, *at4CL3*↑, *cotal1*↑) and IMX198 (□) (*aro3*Δ *ARO4*^*G226S*^*aro10*Δ, *pdc5*Δ, *pdc6*Δ, *atPAL1*↑, *coC4H*↑, *coCPR1*↑, *atCHI1*↑, *atCHS3*↑_,_*coCHS3*↑, *at4CL3*↑, *cotal1*↑). Cultures were grown in shake flasks on synthetic medium containing 20 g·l^-1^ glucose. All cultures were performed in duplicate. Error bar denotes deviation of the mean.

Naringenin chalcone synthase, which catalyzes the formation of chalcone by condensing coumaroyl-CoA with three molecules of malonyl-CoA, is known to be an enzyme with low catalytic activity [46]. The transient accumulation and later reconsumption of coumaric acid in shake flask cultures suggested that reactions downstream of coumaric acid were limiting naringenin production. To test whether the capacity of chalcone synthase was indeed controlling flux through the pathway, two additional copies of the *coCHS3* gene were introduced into strain IMX106 on an episomal plasmid (pUDE188), yielding strain IMX197. The two additional copies resulted in a 2.5 fold increase in naringenin accumulation (134.5 μM) in shake flask cultures (Figure 5A), indicating that *coCHS3* was indeed a limiting step in the naringenin production*.* Additional *coCHS3* copies also caused a decreased production of phloretic acid (Figure 5B), consistent with the hypothesis that phloretic acid production occurs when coumaroyl-CoA cannot be efficiently converted to naringenin.

### Expression of a tyrosine ammonia lyase leads to increased naringenin synthesis

The engineered naringenin production pathway directs carbon via the aromatic amino acid phenylalanine to coumaric acid through phenylalanine ammonia lyase, trans-cinnamate 4-monooxygenase and subsequently to naringenin through the 4-coumaric acid-CoA ligase and chalcone synthase reactions (Figure 1). Deregulation of aromatic amino acid synthesis in *S. cerevisiae* by eliminating feed-back inhibition of DAHP synthase not only increases intracellular phenylalanine levels, but also the intracellular concentration of tyrosine [40]. Since *S. cerevisiae* cannot interconvert tyrosine and phenylalanine, this increased intracellular availability of tyrosine could not be exploited for naringenin production. In other organisms, deamination of tyrosine by tyrosine ammonia lyase does provide an alternative route to coumaric acid. This has previously been shown to increase naringenin production by *E coli* when fed with either glucose or tyrosine [18,20,47]. To investigate the added benefit of the direct conversion of tyrosine into coumaric acid in *S. cerevisiae* IMX197, a *cotal1* gene from *Rhodobacter capsulatus* was expressed. This *R. capsulatus* gene was chosen based on its known high catalytic efficiency for tyrosine [48]. Integration of this yeast codon-optimized *cotal1* gene resulted in increased naringenin production in the resulting strain IMX198 (*aro3*Δ *ARO4*^*G226S*^*aro10*Δ, *pdc5*Δ, *pdc6*Δ, *atPAL1*↑, *coC4H*↑, *coCPR1*↑, *atCHI1*↑, *atCHS3*↑_,_*coCHS3*↑, *at4CL3*↑, *cotal1*↑) (Figure 5B), to a titer of 200 μM in shake-flask cultures. Additionally, the phloretic acid concentrations in this strain again increased compared to the parental strain IMX197 (623,9 ± 11,4 μM and 396,1 ± 6,9 respectively), while the coumaric acid concentrations in cultures of the two strains remained the same.

### Naringenin production in controlled aerobic batch cultures

**Figure 6:**
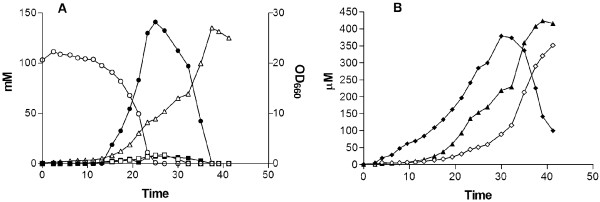
**Product formation of the naringenin-producing strain*S. cerevisiae*IMX198 in bioreactors.** Growth and extracellular metabolite formation were studied in aerobic and pH controlled (pH 5.0) batch cultures of IMX198 on glucose. The results shown are from a single representative experiment. **A**) Concentrations of glucose (○), ethanol (●), acetate (□), glycerol (■) and optical density (OD_660_) (Δ). **B**) Concentrations of naringenin (▲), coumaric acid (♦) and phloretic acid (◊).

To further characterize strain IMX198 (*aro3*Δ *ARO4*^*G226S*^*aro10*Δ, *pdc5*Δ, *pdc6*Δ, *atPAL1*↑, *coC4H*↑, *coCPR1*↑, *atCHI1*↑, *atCHS3*↑_,_*coCHS3*↑, *at4CL3*↑, *cotal1*↑) under controlled conditions, this strain was cultured in a 2L batch bioreactor with 20 g·l^-1^ glucose at pH 5.0 (Figure 6A, B). When *S. cerevisiae* is grown aerobically in batch cultures on glucose, alcoholic fermentation is the predominant mode of glucose metabolism [49,50] and is characterized by a diauxic growth profile. During the glucose consumption phase the specific growth rate of IMX198 was 0.2 h^-1^, which is approximately 50% of the specific growth rate of the reference strain *S. cerevisiae* CEN.PK113-7D under the same conditions [51]. Besides the expected formation of ethanol, a specific naringenin production rate of 12.545 ± 0.333 μmol^.^g^-1^_CDW_h^-1^ was obtained. After complete consumption of glucose, a naringenin titer 148.06 ± 5.67 μM at a naringenin yield on glucose of 0.002 ± 0.000 (g·g^-1^) was obtained. When all the glucose was consumed, ethanol, acetate and glycerol that were produced during the first phase were subsequently consumed (Figure 6A). During this reconsumption phase, naringenin titers increased to 414.63 ± 1.60 μM, indicating that most naringenin is produced during this second phase (Figure 6B). However, it must be taken into account that during this reconsumption phase, naringenin production is also facilitated by the presence of available coumarate that was previously formed during the glucose consumption phase and also by the higher amount of biomass, compared to the glucose phase. When only calculating the product yield over the total glucose and ethanol consumption phase, a yield of 0.006 ± 0.000 (g·g^-1^) was obtained, which is approximately triple of what is obtained during solely the glucose consumption phase.

In summary, the naringenin production of IMX198 in a well aerated and pH controlled batch bioreactor cultivation required only 38 hours and yielded more than twice the naringenin titer that was achieved in equivalent shake flasks cultivations (200 vs. 415 μM).

## Discussion

The present study, for the first time, demonstrates *de novo* production of the key flavonoid intermediate naringenin, from glucose, by an engineered *S. cerevisiae* strain. Combined expression of the product pathway, codon optimization, improvement of precursor supply and reduction of byproduct formation led to concentrations of over 400 μM naringenin in aerobic, glucose-grown batch cultures. These concentrations are over 4-fold higher than reported in a previous study on *de novo* biosynthesis of naringenin by an engineered *E. coli* strain [18].

In previous studies on microbial biotransformation of aromatic precursors to naringenin, the core biosynthetic pathway for naringenin synthesis (phenylalanine and/or tyrosine ammonia lyase, trans-cinnamate 4-monooxygenase, P450 cytochrome reductase, 4-coumaric acid-CoA ligase, chalcone synthase) was, in most cases, reconstituted by co-expressing genes from different plants and/or bacteria [17-19,27,28]. Only one of those studies used naringenin biosynthetic genes originating from a single donor organism (*A. thaliana*), which resulted in the first demonstration of *de novo* naringenin biosynthesis in *E. coli*[20]. In line with this observation, co-expression of a set of biosynthetic genes from *A. thaliana*, that were selected based on correlation of *in planta* expression profiles, was sufficient for low-level *de novo* production of naringenin in *S. cerevisiae* (Figure 3). The observations that heterologous co-expression of genes originating from single plant species is beneficial for naringenin production, support the hypothesis that maintaining donor specific protein-protein interactions can improve pathway kinetics and specificity [52]. Besides using genes from a single plant species, also the recently developed synthetic protein scaffolds may be used to explore the impact of the physical association of pathway enzymes [53-55]. The potential of this approach is illustrated by the recent demonstration that protein scaffolding in yeast led to a five-fold increase of the titer of the stilbenoid resveratrol [55]. The optimized supply of precursors and decreased production of aromatic byproducts make the yeast strains developed in this study an interesting models to explore the impact of pathway topology on flavonoid production in a heterologous host.

The improvement of naringenin production in this study followed a typical metabolic engineering approach consisting of repeated cycles of design, construction, and analysis [56]. After introduction of the pathway-encoding genes and observation of low levels of naringenin, alleviation of feedback inhibition of aromatic amino acid biosynthesis by phenylalanine and tyrosine further improved naringenin production. As seen previously [57]. removal of feedback inhibition also resulted in an overflow of phenylalanine into the Ehrlich pathway [40] and, consequently, production of phenylethanol as a major byproduct. Restricting the activity of the Ehrlich pathway by deletion of three decarboxylase-encoding genes strongly reduced formation of phenylethanol and had a strong positive impact on naringenin production (Figure 3). Precursor supply was further improved by expression of a tyrosine ammonia lyase, which introduced a second route towards coumarate besides phenylalanine ammonia lyase. The subsequent introduction of two additional copies of the *A. thaliana CHS3* gene further increased the flux from coumaric acid to naringenin (Figure 5B). This observation may be attributed to the catalytic properties of *CHS3*. This enzyme combines 4 substrates (coumaroyl CoA and three copies of malonyl CoA) to synthesise naringenin, and it is well known to exhibit a very low catalytic activity. Although originating from a different plant, the catalytic constant kcat of the chalcone synthase *CHS3* from alfalfa is 2 min^-1^, [58], which is roughly 25-fold lower than the kcat of the protein preceding the chalcone synthase in the metabolic pathway, the coumaroyl-CoA ligase (4CL3) from *A.thaliana* which has a kcat of 50 min^-1^[23].

In the naringenin-producing *S. cerevisiae* strains, the by-product phloretic acid was produced in concentrations similar to those achieved for naringenin. Phloretic acid production was only produced in cultures that both accumulated high concentrations of coumaric acid and expressed *A. thaliana 4CL3*. Phloretic acid has previously been detected in engineered *S. cerevisiae*, but the mechanism of its formation remains unknown [59]. Even though it was observed that increasing the flux to naringenin by increasing the copy number of the *A. thaliana CHS3* gene, further analysis of the mechanism of phloretic acid production by these engineered *S. cerevisiae*, e.g. via systematic gene deletion studies, provides a good opportunity to further improve naringenin production. Also improvement of the provision of malonyl-CoA, a key precursor for both naringenin production as well as for lipid production, might further improve naringenin production. It has previously been shown that overexpression of acetyl-CoA carboxylase, which carboxylates acetyl-CoA to malonyl-CoA, leads to increased production of fatty acids in the yeast *Hansenula polymorpha*[60] and of biodiesel or 6-methylsalicylic acid production in *S. cerevisiae*[61,62]. Moreover, malonyl-CoA synthase in *E. coli* combined with malonate supplementation, improved biotransformation of coumaric acid into flavonoids [63]. Increasing the availability of malonyl-CoA therefore provides an interesting target for further research.

Demonstration of *de novo* production of naringenin in *S. cerevisiae* represents an important step towards commercial production of plant-derived flavonoids from glucose. Even though commercial production of naringenin for direct consumer applications will require substantial further improvement of product yields and titers. Furthermore, this engineered naringenin-producing yeast strain provides an attractive platform for expression of plant-derived pathways that convert naringenin into other flavonoids, either by reconstruction of individual plant pathways or by combinatorial approaches based on the powerful yeast-based methods for *in vivo* recombination [29,64]. Availability of a robust yeast platform for such studies paves the way for identification, characterization and eventual industrialization of many plant-derived flavonoids with pharma- and nutraceutical properties.

## Conclusions

Here we provide the first report on the production of the flavonoid naringenin from glucose as sole carbon source using the yeast *S. cerevisiae*, and the optimization of the strain to reach a naringenin titer of over 400 μM. Several obstacles were overcome when expressing a foreign pathway in *S. cerevisiae,* which include the choice of enzymes, the expression systems the optimization of flux and the inhibition of competing pathways. This study successfully describes the reduction of the phenyl ethanol side product formation. All were done to increase the final titer of flavonoid production.

This strain may be used as an ideal platform for production of a wide variety of flavonoids originating from naringenin, thereby facilitating the production, characterization and possible implementation of such compounds as pharmaceutical and neutraceutical.

## Methods

### Strains and maintenance

All strains used in this study (Table 2) are derived from the *S. cerevisiae* CEN.PK strain family background [65,66]. Stock cultures were grown at 30°C in 500 ml shake flask cultures containing 100 ml synthetic medium (according to [67]) with 20 g·l^-1^ glucose and appropriate growth factors to supplement the specific auxotrophic requirements of the strains [68]. After overnight growth, a final concentration of 20% glycerol was added and 1 ml aliquots were stored at -80°C.

**Table 2:** Strains and plasmids used in this study

**Strain**	**Genotype**	**Source**
CEN.PK2-1c	MATalpha *ura3 his3 leu2 trp1* MAL2-8cSUC2	[69]
CEN.PK717.5A	MATa *aro3*Δ *ARO4*^*G226S*^	This study
IMU011	MATalpha *ura3 his3 leu2 trp1 MAL2-8cSUC2*, pUDE172, *PYK2*::pUDI065	This study
IMK328	MATalpha *ura3 his3 leu2 trp1 MAL2-8cSUC2 Δaro3*(46,1065)::*loxP ARO4*^*G226S*^	This study
IMK389	IMK328 *pdc6Δ*(-6,-2)*::loxP pdc5Δ*(-6,-2)*::loxP*	This study
IMK393	IMK389 *aro10Δ*(-6,-2)*::loxP*	This study
IMX183	IMK328 pUDE172, *PYK2*::pUDI065	This study
IMX185	IMK389 pUDE172, *PYK2*::pUDI065	This study
IMX106	IMK393 pUDE172, *PYK2*::pUDI065	This study
IMX193	IMK393 pUDE172, *PYK2:*:pUDI065, *TRP1*::pUDI069	This study
IMX197	IMK393 pUDE172, *PYK2*::pUDI065, pUDE188	This study
IMX198	IMK393 pUDE172, *PYK2*::pUDI065, *TRP1*::pUDI069, pUDE188	This study
**Plasmids**		
pUG6	PCR template for loxp-*KanMX4*-loxp cassette	[70]
pSH47	Centromeric plasmid, *URA3*, P_GAL1_-*Cre-*T_CYC1_	[70]
pAG416GPD-*ccdB*	Centromeric plasmid, *URA3*, P_TDH3_-*ccdb*-T_CYC1,_ Addgene plasmid 14148	[71]
pAG306GPD-*ccdB*	Integration plasmid, *URA3*, P_TDH3_-*ccdb*-T_CYC1,_ Addgene plasmid 14140	[71]
pAG305GPD-*ccdB*	Integration plasmid, *LEU2*, P_TDH3_-*ccdb*-T_CYC1,_ Addgene plasmid 14138	[71]
pAG304GPD-*ccdB*	Integration plasmid, *TRP1*, P_TDH3_-*ccdb*-T_CYC1,_ Addgene plasmid 14136	[71]
pAG423GPD-*ccdB*	2 μm ori, *HIS3*, P_TDH3_-*ccdb*-T_CYC1,_ Addgene plasmid 14150	[71]
p426TEF	2 μm ori, *URA3,* P_TEF_ –T_TEF_	[72]
p426TPI	2 μm ori, *URA3* P_TPI_ –T_ADH_	[73]
pUNI.U21762	Cloning vector, Ka, *atCHS3* cDNA^#^	[74]
pUNI.U11924	Cloning vector, Ka, *atCHI1* cDNA	[74]
pDZ.4CL3	Cloning vector, Bla, *at4CL3* cDNA	[75]
pUD168	pMA cloning vector, Bla, A-P_TDH3_-*atPAL1*-T_CYC1_-B^*^	This study
pUD169	pMA cloning vector, Bla, B-P_PGI_-*coCPR1*-T_PGI_-C	This study
pUD170	pMA cloning vector, Bla, C-P_TPI_-*coC4H*-T_ADH_-D	This study
pUD175	pMA cloning vector, Bla, E-P_PGK_-*coCHS3*-T_PGK_-F	This study
pUDE185	2 μm ori, *HIS3*, P_TDH3_-*coCHS3*-T_CYC1_	This study
pUDE186	2 μm ori, *URA3,* P_TEF_-*coCHS3*-T_TEF_	This study
pUDE188	2 μm ori, *HIS3*, P_TDH3_-*coCHS3*-T_CYC1_, P_TEF_-*coCHS3*-T_TEF_	This study
pUDE103	2 μm ori, *URA3,* P_TEF_-*at4CL3*-T_TEF_	This study
pUDE172	Centromeric plasmid, *URA3*, P_TDH3_-*atPAL1*-T_CYC1_, P_TPI_-*coC4H*-T_ADH,_ P_PGI_-*coCPR1*-T_PGI_	This study
pUDI053	Integration plasmid, *LEU2*, P_TDH3_-*atCHI1*-T_CYC1_	This study
pUDI060	Integration plasmid, *LEU2*, P_TDH3_-*atCHI1*-T_CYC1_, P_TEF_-*at4CL3*-T_TEF_	This study
pUDI061	Integration plasmid, *LEU2*, P_TDH3_-*atCHI1*-T_CYC1_, P_TPI_-*atCHS3*-T_ADH,_ P_TEF_-*at4CL3*-T_TEF_	This study
pUDI065	Integration plasmid, *LEU2*, P_TDH3_-*atCHI1*-T_CYC1_, P_TPI_-*atCHS3*-T_ADH,_ P_TEF_-*at4CL3*-T_TEF_, *PYK2*(1-710)	This study
pUDI069	Integration plasmid, *TRP1*, P_TDH3_-*coTAL1*-T_CYC1_	This study

* *co*: codon optimized, #; *at*: *A. thaliana*.

### Introduction of chromosomal gene alteration

Plasmids and oligonucleotide primers used in this study are listed in Tables 2 and 3, respectively. The geneticin (G418) resistance cassette (*KanMX4)* was amplified by using the pUG6 vector as the template [70]. The *KanMX4* PCR product was transformed to the appropriate host (Table 2) using the lithium acetate method [76]. Synthetic medium agar plates containing 2% glucose and 200 mg·l^-1^ G418 (Sigma-Aldrich, Zwijdrecht, The Netherlands) and appropriate growth factors to supplement the auxotrophic requirements of the strains, were used to select for the presence of the *KanMX4* gene. The *KanMX4* marker was removed by the *CreloxP* recombination system using the plasmid pSH47 as described previously [70].

**Table 3:** Oligonucleotide primers used in this study

**Name**	**Sequence (5’-3’)**
**Primers for knockouts**	
ARO3 KO for	ATGTTCATTAAAAACGATCACGCCGGTGACAGGAAACGCTTGGAAGACTGCAGCTGAAGCTTCGTACG
ARO3 KO rev	CTATTTTTTCAAGGCCTTTCTTCTGTTTCTAACACCTTCTGCCAATAGCTGCATAGGCCACTAGTGGATCTG
PDC5 KO for	CATAATCAATCTCAAAGAGAACAACACAATACAATAACAAGAAGAACAAACAGCTGAAGCTTCGTACGC
PDC5 KO rev	AAAGTAAAAAAATACACAAACGTTGAATCATGAGTTTTATGTTAATTAGCGCATAGGCCACTAGTGGATCTG
PDC6 KO for	GGCGGCTGTTTGAAGCCATTCTATCTTAATCTTGTGCTATTGCAGTCCTCCAGCTGAAGCTTCGTACGC
PDC6 KO rev	GTAAGTTTTATTTGCAACAATAATTCGTTTGAGTACACTACTAATGGCGCATAGGCCACTAGTGGATCTG
ARO10 KO for	GATACTCAAAACAAGTTGACGCGACTTCTGTAAAGTTTATTTACAAGATAACAAAGAAACTCCCTTAAGCCAGCTGAAGCTTCGTACGC
ARO10 KO rev	GGGTTTTTTATGTGTTAATGAACAGAAAACGAACAATTGGTAGCAGTGTTTTATAATTGCGCCCACAAGTCATAGGCCACTAGTGGATCTG
ARO4 for	GACGCATTGTTAGCATTG
ARO4 rev	CGAATTGGCAGTGGTAGAG
**Primers for cloning**	
FK7	GCGACTAGTATGTCTTCATCCAACGCC
FK8	GCGGTCGACTCAGTTCTCTTTGGCTAGTT
FK9	GCGACTAGTATGATCACTGCAGCTCTAC
FK10	GCGGTCGACTCAACAAAGCTTAGCTTTGAG
FK11	GAGGCCGGCGCAATTAACCCTCACTAAAG
FK12	TGGACTCCAACGTCAAAG
FK13	GCGACTAGTATGGTGATGGCTGGTGC
FK14	GCGCTCGAGTTAGAGAGGAACGCTGTGC
FK15	CGCGCAATTAACCCTCAC
FK16	GCGGAGCTCCACTATAGGGCGAATTGGG
FK29	GCGTCCCGGAATGCCAGAGTCCAGATTG
FK30	GCGTCCCGGAGATATCTTGCCCTTCAGAACCC
FK52	GCGACTAGTATGACCTTACAATCCCAAAC
FK53	GCGCTCGAGTTAGGCTGGAG
FK86	ATCATGAACTTGCGCTCAATTCCGCGCAGAAGGCAATGCTATAAGACCTACGTCCACGGATTGCGCCTAAGACCGGATAAAGCACCGCATAGGGTAATAACTG
FK87	ATCGGAAATTCGACCGTGTGCTAGTGCCTATTGATGATCTGGCGGAATGTCTGCCGTGCCATAGCCATGCCTTCACATATAGTGTAATACGGTTATCCACAGAATC
FK169	GCGACTAGTATGGTTATGGCTGGTGC
FK170	GCGCTCGAGAATTACAATGGAACAGAGTGC
FK171	GCGGAGCTCGCTGGAGCTCATAGCTTC
FK172	GCGGAGCTCGTACCCAGTATAGCGACC
**Primers for verification**	
FK105	TCTTTCCTGCGTTATCCC
FK106	GGCATGTACGGGTTACAG
FK107	CGCGTGTACGCATGTAAC
FK108	TCCCGTTAGGAACATTGG
FK109	GCAAATGCCTGCAAATCG
FK110	AACGTGCAGATGGTGATG
FK111	CATTATTGAACAGCGTCCAAG
FK112	AGAACCGTGGATGATGTG

The introduction of a feed-back-insensitive DAHP synthase activity in *S.cerevisiae* was accomplished in two steps. Firstly, the *ARO3* allele was deleted in strain CEN.PK 2.1C using primers ARO3 KO for and ARO3 KO rev as described above. In this resulting strain, the chromosomal *ARO4* allele was replaced by the feed-back-insensitive *ARO4*^*G226S*^ allele [38,39]. Genomic DNA of strain CEN.PK717.5A was prepared using the genomic DNA isolation kit (Zymo Research, Orange, CA), according to the manufacturer’s recommendations. The *ARO4*^*G226S*^ allele was then amplified using primers Aro4 for/Aro4 rev, and transformed. Selection was performed by screening for tyrosine tolerance by plating on selective agar plates containing synthetic medium, 2% glucose and 1 g·l^-1^ tyrosine. The resulting strain was named IMK328 (Table 2).

### Plasmid construction

Plasmid pUDE172 was obtained by transformation-associated recombination methods as described previously [29,30,77]. The backbone fragment harboring the yeast and bacterial origins of replication and markers was amplified from pAG416GPD-*ccdB* (Table 2) using primers FK86/87. Genes were optimized for *S. cerevisiae* using the JCat algorithm [78]), based on the *A. thaliana* gene sequence. The DNA fragments were synthesized (Life Technologies, Bleiswijk, The Netherlands) harboring the yeast codon-optimized genes (codon, promoter, terminator and 2 unique 80 bp flanks that included an *Eco*RV sites at each end). The codon-optimized plant genes were flanked by different promoter and terminator combinations to prevent plasmid instability due to homologous recombination. Prior to transformation-associated recombination, the synthesized fragments were digested from the cloning vector using *Eco*RV and gel purified. The pAG416GPD-*ccdB* backbone fragment and the 3 gene cassettes *atPAL1, coC4H* and *coCPR* were mixed at a ratio of 1:2:2:2 (50ng :100ng; 100ng: 100ng) and transformed to CEN.PK2-1C. Colonies were selected on glucose synthetic medium plates in the absence of uracil and checked by multiplex PCR using the primers FK105 – FK112. After plasmid isolation, retransformation to *E. coli* and subsequent plasmid purification, the structure of the resulting plasmid pUDE172 (Genbank accession number: JX268037) was verified using restriction analysis.

The *A. thaliana CHI1* gene was amplified using primers FK7/8 from plasmid pUNI.U21762 (Table 2), and then agarose gel purified and digested using *Spe*I and *Sal*I. The restricted fragment was ligated into an *Spe*I and *Sal*I linearized pAG305-GPD-*ccdB* plasmid. The *A. thaliana 4CL3* gene was amplified using primers FK9/10 from pDZ.4CL3, purified and then ligated into p426TEF (Table 2) using *Spe*I and *Sal*I. Subsequently, the P_TEF_-at*4CL3*-T_TEF_ cassette was amplified using primer FK11/12, purified and ligated into a *Nae*I digested and FastAP (Thermo Scientific, Etten-Leur, The Netherlands) treated pUDI053, yielding pUDI060. The *A. thaliana CHS3* gene was amplified using primer FK13/14, purified and ligated into p426TPI (Table 2) using *Spe*I and *Xho*I yielding pUDE101. The P_TPI_-*atCHS3*-T_ADH_ cassette was amplified using primer FK15/16, purified and digested by *Sac*I. The resulting fragment was ligated into *Sac*I digested and FastAP treated pUDI060 plasmid, yielding pUDI061. A fragment of the *PYK2* gene (1-710bp) was amplified from the CEN.PK2-1C genome using primer FK29/30, and then purified and digested using *Pfo*I. This fragment was ligated into pUDI061 plasmid that had been digested by *Pfo*I and treated with alkaline phosphatase (FastAP, Thermo Scientific) yielding pUDI065 (Genbank accession number: JX268039). The resulting plasmid was linearized using *Nco*I and integrated into the chromosomal *PYK2* locus.

The *tal1* gene from *R. capsulatus*[48,79], was codon optimized for yeast using JCat [78], and synthesized (GeneScript, Piscataway, NJ). The synthetic *cotal1* was amplified using primers FK52/53 and ligated into pAG304-*ccdB* using *Spe*I and *Xho*I resulting in pUDI069 (Genbank accession number: JX268036). Integration of this plasmid into the *trp1* locus was preceded by linearization using *Eco*RV.

A DNA fragment containing the custom-synthesized, yeast codon-optimized *CHS3* sequence (Life Technologies, Bleiswijk, The Netherlands) was amplified using primers FK169/170 and ligated into both a pAG325GPD-*ccdB* and p426TEF (Table 2) using *Spe*I and *Xho*I yielding respectively, pUDE185 and 186. Subsequently, the P_TEF_-*coCHS3*-T_TEF_ cassette was amplified using primers FK 171/172 and restricted using *Sac*I. This cassette was ligated into a *Sac*I digested and FastAP (Thermo Scientific) treated pUDE185. Plasmids were screened for opposing gene orientation by restriction analysis, yielding pUDE188 (Genbank accession number: JX268038).

### Molecular biology procedures

PCR amplification was performed using Phusion® Hot Start II High Fidelity Polymerase (Thermo Scientific) according to the manufacturer's instructions in a Biometra TGradient Thermocycler (Biometra, Gottingen, Germany). Agarose gel separation was performed using 1% (w/v) agarose (Sigma-Aldrich, Zwijdrecht, The Netherlands) gel in 1×TAE (40 mM Tris-acetate pH 8.0 and 1 mM EDTA). Isolation of agarose trapped fragments was performed using Zymoclean Gel DNA Recovery kit (Zymo Research, Orange, CA). Restriction endonucleases, DNA ligases and FastAP (Thermo Scientific) were used according to the manufacturer's instructions. Transformation and amplification of plasmids were performed in *E. coli* DH5α electrocompetent cells (Invitrogen, Carlsbad, CA) according to the manufacturer's instructions. Plasmids were isolated from *E. coli* with the Sigma GenElute Plasmid Miniprep Kit (Sigma-Aldrich). Alternatively, plasmid purification was performed using a Zymoprep™™ Yeast Plasmid Miniprep (Zymo Research). Sequencing of constructs was performed by Baseclear BV (Baseclear, Leiden, The Netherlands).

### Cultivation and media

*E. coli* was grown at 37°C in Luria Broth medium containing the appropriate antibiotic, 100 μg·l^-1^ ampicillin or 50 μg·l^-1^ kanamycin (Sigma-Aldrich, Zwijndrecht, The Netherlands). *S. cerevisiae* was grown at 30°C in medium containing demineralized water, 20 g·l^-1^ glucose, 5 g·l^-1^ (NH_4_)_2_SO_4_, 3 g·l^-1^ KH_2_PO_4_, 0.5 g·l^-1^ MgSO_4_.7H_2_O, vitamins and trace elements [67]. The pH of the medium was set to 6.0 using KOH. Appropriate growth factors to supplement the auxotrophic requirements of the strains were added at 150 mg·l^-1^ for uracil, 500 mg·l^-1^ for leucine, 75 mg·l^-1^ for tryptophan and 125 mg·l^-1^ for histidine [68]. Unless otherwise stated, cultures were grown in 500 ml shake flasks with 100 ml medium by adding 1 ml frozen stock culture and incubating at 30°C in an Innova incubator shaker (New Brunswick Scientific, Edison, NJ) set at 200 rpm. Controlled aerobic batch cultures were grown at 30°C in 2l bioreactors (Applikon, Schiedam, The Netherlands), using a working volume of 1l. In the bioreactor experiments, the (NH_4_)_2_SO_4_ concentration in the synthetic medium was increased to 10 g·l^-1^ to avoid nitrogen depletion towards the end of the culture. Emulsion C antifoam at 0.05% (w/v) (Sigma-Aldrich, Zwijndrecht, The Netherlands) was added separately after autoclaving. The pH was maintained at pH 5.0 by automatic addition of either 2 M KOH or 2 M H_2_SO_4_, the stirrer speed was fixed at 800 rpm and the aeration rate was set at 500 ml·min^-1^.

### Analytical methods

Optical density was measured at 660 nm using a Libra S11 spectrophotometer (Biochrom, Cambridge, UK). Biomass dry weights were determined by filtration of 10 ml culture over dry, preweighed 0.45 μm nitrocellulose filters (Gelman Laboratory, Ann Arbor, USA). After removal of the medium, the filter was washed twice with demineralized water and dried for 20 min using a microwave set at 350 W. Glucose, ethanol, glycerol and acetate were analyzed using an Aminex HPX-87H ion exchange column (BioRad, Veenendaal, The Netherlands) operated at 60°C with 5 mM H_2_SO_4_ as mobile phase at a flow rate of 0.6 ml·min^-1^. For measurement of phenylpropanoic compounds, culture samples were diluted with an equal volume of 100% ethanol. After vigorous mixing, cells were spun down at 13000 rpm in a Sorval Heraeus #3328 rotor for 2 min. The supernatant was analysed using HPLC. For phenylpropanoic compounds, measurements were made using a Waters 2695 separation module and a Waters 996 photodiode array detector. Phloretic acid, coumaric acid, phenyl ethanol, cinnamic acid and naringenin were measured respectively at 275, 309, 214, 277, 289 nm using an Agilent Zorbax SB-C18 Column (4.6 × 5.0, 3.5 micron) operating at 30^o^C. A gradient of acetonitrile and 20 mM KH_2_PO_4_ (pH 2) with 1% acetonitrile was used as eluent, at a flow rate of 1 ml·min^-1^, increasing from 0 to 10% acetonitrile in 6 min followed by an increase to 40% acetonitrile until 23 min. From 23 min to 27 min, the flow was set to 100% KH_2_PO_4_. Naringenin, coumaric acid, cinnamic acid, phloretic acid and phenylethanol standards were obtained from Sigma Aldrich (Sigma-Aldrich, Zwijndrecht, The Netherlands).

For analysis of carbon dioxide production in bioreactor cultures, the off-gas was first cooled in a condenser (2°C) and dried with a Perma Pure Dryer (Permapure, Toms River, NJ). CO_2_ concentrations in the off-gas were then measured with a NGA 2000 Rosemount gas analyzer (Rosemount Analytical, Orrville,OH).

Identification of phenylpropanoid intermediates and naringenin was performed using liquid chromatography coupled to both photodiode-array detection and accurate mass quadrupole time-of-flight mass spectrometry (LC–PDA-QTOF MS) was performed using a Waters Alliance 2795 HPLC connected to a Waters 2996 PDA detector and subsequently a QTOF Ultima V4.00.00 mass spectrometer (Waters, MS technologies, UK) operating in negative ionization mode, an analytical column (Luna 3 μ C18/2 100A; 2.0 × 150 mm) attached to a C18 pre-column (2.0 × 4 mm; AJO-4286; both Phenomenex, USA). Eluents A (ultrapure water:formic acid (1000:1, v/v) ) and B (acetonitrile : formic acid (1000 : 1, v/v) ) were used at 0.19 ml.min. The gradient started at 5% B and increased linearly to 35% B in 45 min, after which the column was washed and equilibrated for 15 min before the next injection. The injection volume was 5 μl. Leucine enkephalin ([M-H]- = 554.2620) was used as a lock mass for on-line accurate mass correction [80]. Data were recorded using MassLynx 4.0 software (Waters).

### Co-expression correlation analysis of naringenin biosynthetic genes in *A. thaliana*

Expression correlation analysis was performed using the BAR Expression Angler [35]. Each candidate gene listed in Table 1 was used as “bait”, and expression was correlated over a set of 392 micro-array experiments generated using the ATH1 Affymetrix Whole Genome GeneChip, from the Nottingham Arabidopsis Stock Centre's microarray database [34]. Correlations between expression levels of genes from Table 1 were recorded when the Pearson correlation coefficient was above 0.6.

## Competing interests

The authors declare that they have no competing interests.

## Authors’ contributions

FK, AJAM, JTP and JB designed the study. FK engineered the plasmids, strains and all genetic alterations and performed batch fermentations. FK and BC performed all shake flask culture experiments. JB and AvdH performed the transcriptional correlation analysis. FK performed all analytical quantification with the input of JB. FK, JB, AJAM, JTP, RDH, DB and J-MD drafted the manuscript. All authors have critiqued and approved the final manuscript.
